# Stress hyperglycemia may have higher risk of stroke recurrence than previously diagnosed diabetes mellitus

**DOI:** 10.18632/aging.202797

**Published:** 2021-03-22

**Authors:** Yuzhou Guo, Guangyao Wang, Jing Jing, Anxin Wang, Xiaoli Zhang, Xia Meng, Xingquan Zhao, Liping Liu, Hao Li, David Wang, Yongjun Wang, Yilong Wang

**Affiliations:** 1 Department of Neurology, Beijing Tiantan Hospital, Capital Medical University, Beijing, China; 2 China National Clinical Research Center for Neurological Diseases, Beijing, China; 3 Center of Stroke, Beijing Institute for Brain Disorders, Beijing, China; 4 Beijing Key Laboratory of Translational Medicine for Cerebrovascular Disease, Beijing, China; 5 Department of Neurology, The Affiliated Hospital of Inner Mongolia Medical University, Hohhot, China; 6 Neurovascular Division, Department of Neurology, Barrow Neurological Institute, St. Joseph's Hospital and Medical Center, Phoenix, AZ 85013, USA

**Keywords:** diabetes mellitus, stress hyperglycemia, stroke, TIA, prognosis

## Abstract

We aim to evaluate the risk of stroke recurrence among non-diabetes mellitus (non-DM), previously diagnosed diabetes mellitus (PDDM), newly diagnosed diabetes mellitus-related hyperglycemia (NDDM-RH) and stress hyperglycemia after minor stroke or TIA. Totally, 3026 patients with baseline fasting glucose and glycated albumin from the CHANCE trial (Clopidogrel in High-Risk Patients with Acute Nondisabling Cerebrovascular Events) were included. Patients were classified as non-DM, PDDM, NDDM-RH and stress hyperglycemia according to the status of glucose metabolism. The primary outcome was stroke recurrence during 90-day follow up. Cox regression was performed to estimate the relationship between the status of glucose metabolism and risk of 90-day stroke recurrence. Compared with PDDM, NDDM-RH had a similar risk of 90-day stroke recurrence (hazard ratios [HR]1.39, 95% confidence intervals [CI] 0.94-2.04), while stress hyperglycemia had approximately a 5.3-fold increased risk of 90-day stroke recurrence after adjusted for confounding covariates (HR 5.32, 95% CI 3.43-8.26). Parallel results were found for 90-day recurrent ischemic stroke and composite events. Compared with PDDM in minor stroke or TIA, a parallel risk of 90-day stroke recurrence were observed for NDDM-RH, while stress hyperglycemia might relate to higher risk of 90-day stroke recurrence.

## INTRODUCTION

Minor stroke and transient ischemic attack (TIA) comprises 65% among acute ischemic cerebrovascular diseases. [1] Patients with TIA or minor stroke are related to higher stroke risk after symptom onset at the early period. [2, 3] Although intensified dual antiplatelet therapy, a small portion of patients with minor stroke or TIA still have recurrent stroke. [4–6] Diabetes mellitus (DM) has been demonstrated as a independent predictor of stroke recurrence after index events of ischemic stroke or TIA. [7–10] Patients with DM have a worse vascular prognosis than nondiabetic patients. [11] Furthermore, DM have been widely applied for predicting outcomes after acute ischemic stroke or TIA. [12, 13] Consequently, the presence of pre-existing DM have received much attention in stroke patients. Newly diagnosed diabetes mellitus-related hyperglycemia (NDDM-RH) can predict one-year stroke recurrence, death, and poor functional outcome compared with non-diabetes mellitus (non-DM) in ischemic stroke. [14] Stress hyperglycemia has been identified as a predictor of stroke recurrence and poor outcome after TIA or ischemic stroke. [15, 16] A previous study has shown that NDDM was associated with higher risk of death [17] and more likely to have poorer functional outcome and more severe strokes than patients with previously diagnosed diabetes mellitus (PDDM). [18] However, very few studies have compared the risk of recurrent stroke among non-DM, PDDM, NDDM-RH and stress hyperglycemia after minor stroke or TIA. Previous studies have shown that patients with poor glucose control or diabetes mellitus were less sensitive to aspirin [19, 20] and the interaction of glucose metabolism status according to therapy of aspirin only or combination of clopidogrel and aspirin is uncertain.

We aim to investigate the associations of non-DM, PDDM, NDDM-RH and stress hyperglycemia with outcomes in patients with minor stroke or TIA from the Clopidogrel in High-risk patients with Acute Nondisabling Cerebrovascular Events (CHANCE) trial. In addition, we aim to evaluate the interaction effect of different glucose metabolism status by treatment of aspirin only or combination of clopidogrel and aspirin after minor stroke or TIA.

## RESULTS

### Baseline characteristics

Among the 3044 consecutive patients enrolled in the prespecified biomarker substudy of CHANCE, 3026 (99.4%) patients with available fasting blood glucose and glycated albumin (GA) were included in this subanalysis. The median age of the total patients was 62.2 years and 66.5% of them were men. The median fasting blood glucose level was 5.5 mmol/L (interquartile, 4.9-6.5 mmol/L).

Among the 3026 patients, 2128 (70.3%), 611 (20.2%), 225 (7.4%), and 62 (2.0%) were identified as non-DM, PDDM, NDDM-RH and stress hyperglycemia, respectively. There were 2 (0.09%) patients with type 1 DM in the PDDM. Patients with PDDM had higher proportion of history of ischemic stroke, hypercholesterolemia, myocardial infarction and angina. Patients with NDDM-RH had a higher NIHSS score at admission. Patients with stress hyperglycemia were younger, and had higher proportion of male, overweight, smokers, history of hypertension, and had a TIA as the qualifying event (Table 1). The medians and interquartile ranges of fasting blood glucose were 5.2 mmol/L (4.7-5.7 mmol/L), 7.7 mmol/L (6.1-9.9 mmol/L), 7.9 mmol/L (7.2-10.3 mmol/L), and 7.0 mmol/L (5.6-7.4 mmol/L) for patients with non-DM, PDDM, NDDM-RH, stress hyperglycemia, respectively (p < 0.001) (Supplementary Figure 1). The medians and interquartile ranges of GA were 15.6% (14.5-17.3%), 23.9 % (19.3-30.5%), 21.9% (18.0-29.0%), and 14.1% (13.2-14.8%) for patients with non-DM, PDDM, NDDM-RH, stress hyperglycemia, respectively (p < 0.001) (Supplementary Figure 1).

**Table 1:** Baseline characteristics of the patients by glucose metabolism status.

**Variables**	**Non-DM,** **(n=2128)**	**PDDM,** **(n=611)**	**NDDM-RH, (n=225)**	**Stress hyperglycemia,** **(n=62)**	**p value**
Age, y, median (IQR)	61.8 (54.2-70.9)	63.6 (56.3-71.9)	61.5 (55.5-72.9)	60.0 (52.5-66.8)	0.004
Male, n (%)	1459 (68.6)	364 (59.6)	140 (62.2)	48 (77.4)	<0.001
Body mass index (kg/m2), median (IQR)	24.4 (22.5-26.4)	25.1 (23.4-26.8)	24.8 (22.9-27.0)	25.5 (23.5-27.8)	<0.001
Hs-CRP (mg/L), median (IQR)	1.6 (0.8-3.9)	2.0 (0.9-4.5)	2.3 (1.1-5.8)	1.9 (1.0-4.5)	<0.001
Medical history, n (%)					
Ischemic stroke	385 (18.1)	143 (23.4)	39 (17.3)	11 (17.7)	0.03
TIA	57 (2.7)	27 (4.4)	8 (3.6)	2 (3.2)	0.18
Myocardial infarction	24 (1.1)	27 (4.4)	3 (1.3)	0 (0)	<0.001
Known atrial fibrillation or flutter	41 (1.9)	11 (1.8)	5 (2.2)	0 (0)	0.71
Angina	58 (2.7)	32 (5.2)	3 (1.3)	1 (1.6)	0.005
Valvular heart disease	8 (0.4)	1 (0.2)	1 (0.4)	0 (0)	0.81
Hypertension	1332 (62.6)	443 (72.5)	149 (66.2)	48 (77.4)	<0.001
Hypercholesterolemia	202 (9.5)	94 (15.4)	16 (7.1)	5 (8.1)	<0.001
Current or previous smoking, n (%)	960 (45.1)	228 (37.3)	77 (34.2)	30 (48.4)	<0.001
Time to randomization, n (%)					0.06
<12 hours	1066 (50.1)	311 (50.9)	92 (40.9)	32 (51.6)	
≥ 12hours	1062 (49.9)	300 (49.1)	133 (59.1)	30 (48.4)	
NIHSS on admission, median (IQR)	1 (0-2)	2 (1-2)	2 (1-3)	2 (0-2)	0.01
Qualifying event, n (%)					0.003
TIA	584 (27.4)	160 (26.2)	42 (18.7)	25 (40.3)	
Minor stroke	1544 (72.6)	451 (73.8)	183 (81.3)	37 (59.7)	
Group, n (%)					0.97
Aspirin only	1066 (50.1)	308 (50.4)	116 (51.6)	30 (48.4)	
Clopidogrel + aspirin	1062 (49.9)	303 (49.6)	109 (48.4)	32 (51.6)	
Medications, n (%)					
Antihypertensive	783 (36.8)	235 (38.5)	80 (35.6)	19 (30.6)	0.60
Lipid-lowering	881 (41.4)	258 (42.2)	95 (42.2)	23 (37.1)	0.88

Abbreviations: non-DM, non-diabetes mellitus; PDDM, previously diagnosed diabetes mellitus; NDDM-RH, newly diagnosed diabetes mellitus-related hyperglycemia; TIA: transient ischemic attack; IQR: interquartile range; hs-CRP: high-sensitive C-reactive protein; NIHSS: National Institutes of Health Stroke Scale.

### Clinical outcomes

Overall, 299 (9.9%) patients in this subgroup analysis developed stroke recurrence during 90-day follow up. Among all patients with 90-day stroke recurrence, 151 (7.1%), 80 (13.1%), 39 (17.3%), and 29 (46.8%) were patients with non-DM, PDDM, NDDM-RH and stress hyperglycemia, respectively (Table 2). No obvious interaction effect of mono-antiplatelet or dual-antiplatelet therapy among the four groups were found for the risk of 90-day stroke recurrence (p _interaction_= 0.15, Table 3).

**Table 2:** Risk of outcomes within 90 days after minor stroke or TIA by glucose metabolism status.

**Outcomes**	**Non-DM**	**PDDM**	**NDDM-RH**	**Stress hyperglycemia**
Stroke				
Events, n (%)	151 (7.1)	80 (13.1)	39 (17.3)	29 (46.8)
Adjusted HR (95% CI)^*^	Ref	1.85 (1.41-2.43)	2.61 (1.83-3.71)	9.97 (6.68-14.88)
Adjusted HR (95% CI)^#^	Ref	1.81 (1.38-2.39)	2.51 (1.76-3.59)	9.66 (6.39-14.59)
Adjusted HR (95% CI)^*^	0.54 (0.41-0.71)	Ref	1.41 (0.96-2.07)	5.39 (3.51-8.28)
Adjusted HR (95% CI)^#^	0.55 (0.42-0.73)	Ref	1.39 (0.94-2.04)	5.32 (3.43-8.26)
Ischemic stroke				
Events, n (%)	147 (6.9)	78 (12.8)	39 (17.3)	29 (46.8)
Adjusted HR (95% CI)^*^	Ref	1.85 (1.41-2.44)	2.68 (1.88-3.81)	10.18 (6.82-15.20)
Adjusted HR (95% CI)^#^	Ref	1.81 (1.37-2.40)	2.60 (1.82-3.71)	9.96 (6.59-15.06)
Adjusted HR (95% CI)^*^	0.54 (0.41-0.71)	Ref	1.45 (0.98-2.12)	5.50 (3.57-8.47)
Adjusted HR (95% CI)^#^	0.55 (0.42-0.73)	Ref	1.43 (0.97-2.12)	5.50 (3.54-8.54)
Composite events^‡^				
Events, n (%)	151 (7.1)	81 (13.3)	40 (17.8)	29 (46.8)
Adjusted HR (95% CI)^*^	Ref	1.88 (1.43-2.46)	2.69 (1.89-3.81)	9.97 (6.68-14.87)
Adjusted HR (95% CI)^#^	Ref	1.84 (1.40-2.43)	2.59 (1.82-3.69)	9.66 (6.39-14.58)
Adjusted HR (95% CI)^*^	0.53 (0.41-0.70)	Ref	1.43 (0.98-2.09)	5.31 (3.46-8.15)
Adjusted HR (95% CI)^#^	0.54 (0.41-0.72)	Ref	1.41 (0.96-2.06)	5.24 (3.38-8.13)
Any bleeding				
Events, n (%)	43 (2.0)	14 (2.3)	1 (0.4)	1 (1.6)
Adjusted HR (95% CI)^*^	Ref	1.13 (0.62-2.08)	0.24 (0.03-1.71)	1.40 (0.19-10.17)
Adjusted HR (95% CI)^#^	Ref	1.28 (0.68-2.39)	0.26 (0.04-1.93)	1.57 (0.21-11.65)
Adjusted HR (95% CI)^*^	0.88 (0.48-1.62)	Ref	0.21 (0.03-1.59)	1.23 (0.16-9.43)
Adjusted HR (95% CI)^#^	0.78(0.42-1.46)	Ref	0.21 (0.03-1.58)	1.23 (0.16-9.53)

Abbreviations: non-DM, non-diabetes mellitus; PDDM, previously diagnosed diabetes mellitus; NDDM-RH, newly diagnosed diabetes mellitus-related hyperglycemia; hs-CRP: high-sensitive C-reactive protein. ^*^adjusted for age and sex. ^#^adjusted for age, sex, body mass index, hs-CRP, history of ischemic stroke, TIA, myocardial infarction, known atrial fibrillation or flutter, angina, valvular heart disease, hypertension and hypercholesterolemia, smoking status, NIHSS score on admission, qualifying events of minor stroke or TIA, randomized treatment of aspirin alone or clopidogrel plus aspirin, use of antihypertensive medications, and statin medications. ^‡^Composite events: stroke, myocardial infarction, or death from cardiovascular causes.

**Table 3:** Risk of stroke recurrence within 90 days for clopidogrel-aspirin combined therapy compared with aspirin alone.

**Glucose metabolism status**	**Aspirin**		**Clopidogrel-aspirin**		Model 1^*^			**Model 2^#^**		
	**No.**	**Events, n (%)**	**No.**	**Events, n (%)**	Adjusted HR (95% CI)^*^	**p value**	**p value for interaction**	**Adjusted HR (95% CI)^#^**	**p** **value**	**p value for interaction**
Non-DM	1066	91 (8.5)	1062	60 (5.6)	0.66 (0.47-0.91)	0.01	0.24	0.65 (0.47-0.91)	0.01	0.15
PDDM	308	48 (15.6)	303	32 (10.6)	0.66 (0.42-1.04)	0.07		0.64 (0.40-1.01)	0.05	
NDDM-RH	116	21 (18.1)	109	18 (16.5)	0.90 (0.48-1.70)	0.75		0.98 (0.49-1.95)	0.96	
Stress hyperglycemia	30	21 (70.0)	32	8 (25.0)	0.35 (0.15-0.79)	0.01		0.28 (0.10-0.76)	0.01	

Abbreviations: non-DM, non-diabetes mellitus; PDDM, previously diagnosed diabetes mellitus; NDDM-RH, newly diagnosed diabetes mellitus-related hyperglycemia; hs-CRP: high-sensitive C-reactive protein. ^*^adjusted for age and sex. ^#^adjusted for age, sex, body mass index, hs-CRP, history of ischemic stroke, TIA, myocardial infarction, known atrial fibrillation or flutter, angina, valvular heart disease, hypertension and hypercholesterolemia, smoking status, NIHSS score on admission, qualifying events of minor stroke or TIA, randomized treatment of aspirin alone or clopidogrel plus aspirin, use of antihypertensive medications, and statin medications.

**Figure 1:**
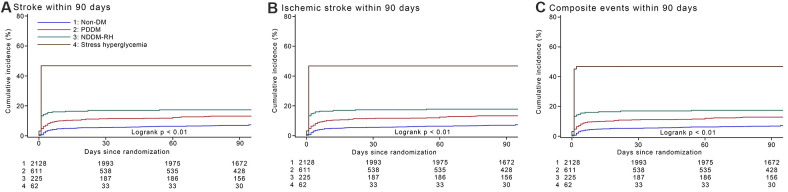
**Cumulative incidence of stroke recurrence, recurrent ischemic stroke and composite events by glucose metabolism status within 90 days.** (**A**).Cumulative incidence of stroke recurrence by glucose metabolism status within 90 days. (**B**) Cumulative incidence of recurrent ischemic stroke by glucose metabolism status within 90 days. (**C**) Cumulative incidence of composite events by glucose metabolism status within 90 days. Abbreviations: non-DM, non-diabetes mellitus; PDDM, previously diagnosed diabetes mellitus; NDDM-RH, newly diagnosed diabetes mellitus-related hyperglycemia.

Compared to patients with non-DM, those with PDDM had a 1.8-times risk of 90-day stroke recurrence (hazard ratios [HR]1.81, 95% confidence interval [CI] 1.38-2.39, p < 0.001), NDDM-RH had a 2.5-times risk of 90-day stroke recurrence (HR 2.51, 95% CI 1.76-3.59, p < 0.001) and stress hyperglycemia had a 9.7-fold risk of 90-day stroke recurrence (HR 9.66, 95% CI 6.39-14.59, p < 0.001), after adjusted for potential covariates. Compared with PDDM, NDDM-RH shown a similar risk of 90-day stroke recurrence (HR 1.39, 95% CI 0.94-2.04, p = 0.10), while stress hyperglycemia had approximately a 5.3-fold risk of 90-day stroke recurrence (HR 5.32, 95% CI 3.43-8.26, p < 0.001), after adjusted for potential covariates. Parallel associations were showed for 90-day recurrent ischemic stroke and composite events. However, no safety concern was observed among the four groups by their glucose metabolism status (Table 2). Cumulative hazards of 90-day outcomes are shown in the Figure 1.

## DISCUSSION

We found that compared with patients with non-DM, those with PDDM, NDDM-RH and stress hyperglycemia were related to higher risk of 90-day stroke recurrence after minor stroke or TIA in this subanalysis of the CHANCE trial. Compared with PDDM, NDDM-RH had a similar risk of 90-day stroke recurrence, while stress hyperglycemia was related to higher risk of 90-day stroke recurrence after minor stroke or TIA. Parallel associations were observed for 90-day recurrent ischemic stroke and composite events.

A cross-sectional study across China of 98658 individuals in 2010 has demonstrated that the prevalence of undiagnosed DM was 8.1%. [21] A hospital-based prospective cohort has indicated that 9.4% of stroke patients had NDDM according to random blood glucose levels>11.1mmol/L or fasting blood glucose levels>7.0 mmol/L and hemoglobin A1c (HbA1c) of ≥6.5 %. [18] After excluding patients with stress hyperglycemia from new detected hyperglycemia during hospitalization, a 7.4 % prevalence of NDDM-RH in this analysis may accurately reflect the prevalence of undiagnosed DM in minor stroke or TIA. Stress hyperglycemia usually refers to transient hyperglycemia during acute illness. However, there is no guidelines specifically definition of stress hyperglycemia and the identification of such patients is complex. Because a part of patients without a history of DM and with fasting blood glucose >7mmol/L or hypoglycemic agents were classified as NDDM-RH in our study, the prevalence of stress hyperglycemia was lower than other studies. [22].

The proportion of type 1 DM was 0.09% among patients with PDDM in our study. A population-based registry study has shown that incidence per 100000 persons years of type 1 DM was 2.68 for 10-14 years with a peak and 0.69 for ≥30 years. The incidence of type 1 DM decreased steadily with age. [23] Another cohort study enrolled Chinese adults aged from 35 to 74 years has shown that the incidence of type 2 DM was 9.6 and 9.2 per 1000 persons years for men and women, respectively. [24] Therefore, the rate of NDDM-RH caused by type 1 DM may be very low in our study, compared to those caused by type 2 DM.

DM has been verified as a distinct risk factor of recurrent stroke after ischemic stroke or TIA. [7–10, 25] A previous study has revealed that NDDM-RH in Chinese patients with acute ischemic stroke was related to 1-year poor outcome. [14] PDDM and NDDM-RH were related to elevated risk of 90-day stroke recurrence in minor stroke or TIA in this study, which was in keeping with the previous research. [14] Patients with NDDM-RH have a similarly poor prognosis in acute myocardial infarction underwent an operation of percutaneous coronary intervention as those with PDDM. [26] Stress hyperglycemia has been demonstrated as a distinct risk factor for in-hospital death and 90-day stroke recurrence after ischemic stroke. [15, 16] However, little research has compared the risk of 90-day stroke recurrence between PDDM, NDDM-RH, and stress hyperglycemia after minor stroke and TIA. Our study added evidence that compared with PDDM, NDDM-RH had a similar risk of 90-day stroke recurrence, while stress hyperglycemia had higher risk of 90-day stroke recurrence after minor stroke and TIA.

These results in our study were conflict with a previous study which demonstrated that in acute ischemic stroke stress hyperglycemia not distinctly associated with unfavorable outcome. [22] Acute ischemic stroke has traditionally been classified as five etiological subtypes based upon the trial of ORG 10172 in acute stroke treatment (TOAST) criteria: 1) cardioembolism, 2) large-artery atherosclerosis, 3) small-vessel occlusion, 4) stroke of other determined, and 5) stroke of undetermined. [27] Different subtypes of stroke indicate various etiologies and different clinical prognosis. [28, 29] Compared with the previous study, the CHANCE trial excluded cardioembolic stroke and only enrolled non-cardioembolic high-risk TIA or minor stroke within first 24 hours. [6] Our study population was substantially different from the previous study in terms of etiology of stroke and severity of stroke. Therefore, we infer that specific population in the CHANCE trial may cause the discrepancy.

Compared with PDDM, NDDM-RH had a similar risk of 90-day stroke recurrence after minor stroke or TIA, which may be attributed to several reasons. First, we hypothesize that the most important reason is the low awareness of DM in China. [21] The proportion of patients with diabetes knowing of their diabetes is only 40 % in patients aged 60 years or older. [30] Diabetes untreated for long periods may cause severe vascular damage. Second, the levels of fasting plasma glucose were similar in patients with NDDM-RH and PDDM in our study. Third, patients with PDDM and NDDM-RH tended to have higher levels of hs-CRP, which might be a potential cause for the similar higher risk of 90-day stroke recurrence in PDDM and NDDM-RH after minor stroke or TIA. [31].

Several aspects may account for the phenomenon that stress hyperglycemia had higher risk of 90-day stroke recurrence compared to PDDM after minor stroke or TIA. Firstly, stress hyperglycemia is relative hyperglycemia at risk of critical illness caused by neuro-hormonal derangements and inflammatory response. [32] Secondly, compared with chronic sustained hyperglycemia, fluctuations of blood glucose has a more specific striking impact on oxidative stress [33] and impairs endothelial function. [34] Acute hyperglycemia can increase circulating cytokine concentrations by an oxidative mechanism. [35] These are the critical factors that contribute to cerebral vascular events. Furthermore, a general population has shown that fluctuation of fasting blood glucose significantly increased the risk of cardiovascular diseases in the general individuals. [36] However, future large-scale cohorts are needed to explain this association.

We found that clopidogrel and aspirin was related to lower risk of 90-day stroke recurrence in non-DM and stress hyperglycemia compared with aspirin only, and these associations were not observed in patients with PDDM and NDDM-RH. However, no interaction effect of antiplatelet therapy among the four groups for the risk of 90-day stroke recurrence was observed. This may be ascribed to the small sampling size of our analysis. Caution is still required in our interpretations for lack of follow-up of dynamic glucose and glycated albumin. Future large-scale studies are needed to illustrate the interaction of antiplatelet therapy by different status of glucose metabolism status.

This study had several limitations. First, since we didn’t measure oral glucose tolerance tests and HbA1c, NDDM-RH was based upon fasting blood glucose, GA, and using of hypoglycemic medications during hospitalization, which may have led to misclassification of the groups. Second, patients with self-reported history of DM diagnosed by physician at admission were defined as PDDM. Data from self-reported information was not as precise as data from detailed medical records. Nevertheless, our well-designed randomized controlled trial may make up for this deficiency to some extent. Third, the dynamic changes of GA and fasting blood glucose were not available in the CHANCE trial. Future studies with dynamic changes of those biomarkers are needed. Fourth, patients enrolled in the CHANCE was confined to acute non-cardioembolic high-risk TIA or minor stroke within 24 hours. Our findings may be not inapplicable to other subtypes or moderate to severe acute ischemic stroke. Hence, the results of our study should be interpreted carefully and are needed to be confirmed in the future large-scale studies.

In conclusion, our study demonstrated that PDDM, NDDM-RH and stress hyperglycemia were related to higher risk of 90-day stroke recurrence in minor stroke or TIA. Compared with PDDM, NDDM-RH had a similar risk of 90-day stroke recurrence, while stress hyperglycemia was related to higher risk of 90-day stroke recurrence in minor stroke or TIA. Early identification and rigid management of NDDM-RH and stress hyperglycemia may help to decrease the 90-day stroke risk after minor stroke or TIA.

## MATERIALS AND METHODS

### Study design and population

Description regarding the rationale and design of the CHANCE trial have been reported in detail. [6, 37] Briefly, it was a randomized, double-blind, placebo-controlled clinical trial carried out in 114 hospitals in China from October 1, 2009 to July 30, 2012. In total, 5170 patients with non-cardioembolic minor stroke or high-risk TIA within 24 hours were randomly assigned to the treatment regimens of aspirin only or clopidogrel plus aspirin. Inclusion criteria for the CHANCE trial are summarized as follows: 1) at least 40 years of age; 2) having an acute minor ischemic stroke (NIHSS ≤ 3) or high-risk TIA (ABCD^2^ ≥ 4); 3) able to take study medications within 24 hours after onset. There were 73 (64%) prespecified hospitals took part in the biomarker substudy on a voluntary basis. This biomarker substudy of CHANCE consecutively recruited 3044 patients.

Ethics approval was granted by the Ethics Committee all participating sites. All patients or their representatives provided written informed consent. CHANCE was registered with ClinicalTrials.gov (Number: NCT00979589).

### Data collection

Patient baseline information including age, sex, height, weight, history of DM, hypertension, smoking status, hypercholesterolemia, ischemic stroke, TIA, atrial fibrillation or flutter, myocardial infarction, coronary heart disease, angina, and NIHSS at admission were recorded by trained and certified interviewers masked to randomization by means of face-to-face interviews. Plasma glucose measurements after overnight fasting were performed within 48 hours after admission.

### Measurement of GA and hs-CRP

Fasting venous blood was collected from each fasting patient participating in the biomarker substudy within 24±12 hours after randomization. Blood samples were collected by face-to-face interviews at each center and delivered through cold-chain to Beijing Tiantan Hospital and stored at −80° C. GA assay was centrally measured with a specific equipment (catalog number 4085-717; Ruiyuan Bio-Technique Co.Ltd., Ningbo, China) through a Roche Modular P800 system. We used the percentage of total serum albumin to express the levels of GA. [38, 39] Hs-CRP was measured through a turbidimetric immunoassay (Ji’en Technique Co Ltd, Shanghai, China) on a Roche Modular P800 system (Roche, Basel, Switzerland). [31] All measurements were centrally conducted by laboratory technicians who were not informed of study assignments and clinical outcomes of patients.

### Groups according to glucose metabolism status

Non-DM were defined if patients met all of the following criteria: (1) without a history of physician-diagnosed DM; (2) fasting plasma glucose < 7.0 mmol/L; (3) without using hypoglycemic medications during hospitalization. PDDM was defined based on the self-reported history of physician-diagnosed DM. According to a GA level of ≥15.5% was the optimal cut point that may predict the presence of early-stage diabetes, [40] patients without a history of physician-diagnosed DM but with fasting plasma glucose≥ 7.0 mmol/L, or used medications to decrease blood glucose levels for any reason during hospitalization were classified as NDDM-RH or stress hyperglycemia. NDDM-RH was identified if patients without a history of DM fulfilled these two inclusion criteria: (1) using medications to decrease blood sugar levels for any reason during hospitalization, or fasting plasma glucose≥ 7.0 mmol/L; [8, 41] (2) GA level of ≥15.5%. Stress hyperglycemia was identified if patients without a history of DM fulfilled these two inclusion criteria: (1) using medications to decrease blood sugar levels for any reason during hospitalization, or fasting plasma glucose≥ 7.0 mmol/L; [8, 41] (2) GA level of <15.5%.

### Follow-up and outcome assessment

Patients were followed up by trained site coordinators at 90 days. [6] The primary efficacy outcome was a 90-day stroke recurrence (including ischemic or hemorrhagic stroke). The secondary efficacy outcomes were 90-day recurrent ischemic stroke and composite events (including ischemic stroke, hemorrhagic stroke, myocardial infarction, or vascular causes of death). Safety outcome was any bleeding during 90-day follow up. Any event related to the outcomes was are adjudicated by the central adjudication committee who were not informed of the study treatment assignments.

### Statistical analysis

Categorical variables were expressed as frequencies (percentage) and continuous variables were expressed as medians (interquartile ranges). Categorical variables were estimated by χ^2^ test. Continuous variables were estimated with Kruskal-Wallis test. To estimate the interaction effect of glucose metabolism status by treatment assignments on the 90-day stroke recurrence, we analyzed different glucose metabolism status × treatment assignment on incident of 90-day stroke recurrence by using multivariable Cox models. We performed multivariable Cox regression models to estimate the relationship between different glucose metabolism status and outcomes. Two models were performed. In the first model, age and sex were adjusted. In the second model, all the baseline variables listed in the Table 1 were adjusted. Adjusted hazard ratios (HR) with 95 % confidence intervals (CI) were reported. Cumulative probability of 90-day stroke recurrence, recurrent ischemic stroke and composite events were constructed by Kaplan-Meier curves.

All tests were two-sided and we considered a p value less than 0.05 as statistically significance. Data analysis were done with SAS software, version 9.4 (SAS Institute Inc., Cary, NC).

## Supplementary Material

Supplementary Figure 1
