# Supplementary material for: Stress hyperglycemia may have higher risk of stroke recurrence than previously diagnosed diabetes mellitus
Source: Aging (Albany NY). 2021 Mar 22;13(6):9108–18. doi: 10.18632/aging.202797 (PMC8034909; doi:10.18632/aging.202797)
Supplement: Supplementary Figure 1 [file aging-13-202797-s001.pdf]

SUPPLEMENTARY FIGURE

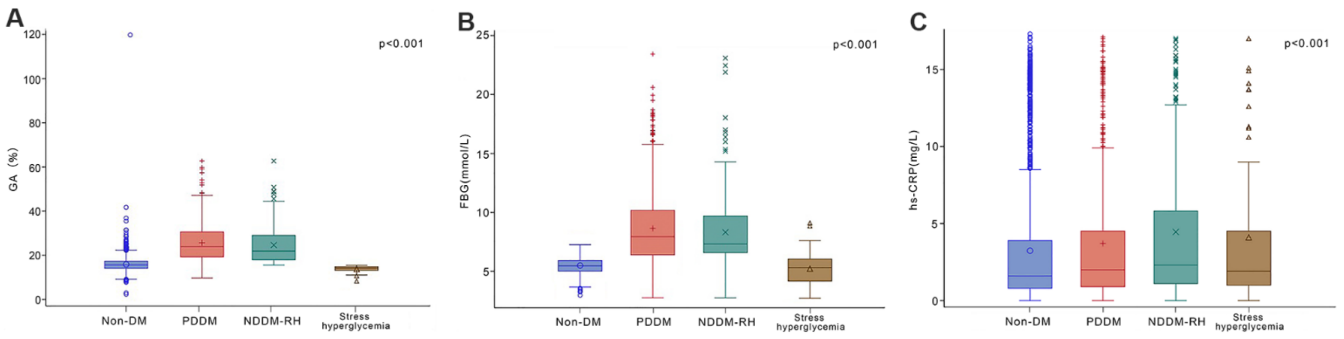

Supplementary Figure 1. Box-plots for levels of GA, FBG and hs-CRP among non-DM, PDDM, NDDM-RH and stress hyperglycemia, respectively. (**A**) Box-plots for levels of GA among non-DM, PDDM, NDDM-RH and stress hyperglycemia. (**B**) Box-plots for levels of FBG among non-DM, PDDM, NDDM-RH and stress hyperglycemia. (**C**) Box-plots for levels of hs-CRP among non-DM, PDDM, NDDM-RH and stress hyperglycemia. Abbreviations: GA indicates glycated albumin; FBG, fasting blood glucose; hs-CRP: high-sensitive C-reactive protein; non-DM, non-diabetes mellitus; PDDM, previously diagnosed diabetes mellitus; NDDM-RH, newly diagnosed diabetes mellitus-related hyperglycemia.
